# Heavy Metal Pollution of Chari River Water during the Crossing of N’Djamena (Chad)

**DOI:** 10.3390/toxics5040026

**Published:** 2017-10-12

**Authors:** N’garam Nambatingar, Yohann Clement, Alain Merle, Tchadanaye New Mahamat, Pierre Lanteri

**Affiliations:** 1 Univ Lyon, CNRS, Université Claude Bernard Lyon 1, ENS de Lyon, Institut des Sciences Analytiques, UMR 5280, 5 rue de la Doua, F-69100 Villeurbanne, France; ngaramn@yahoo.fr (N.N.); yohann.clement@univ-lyon1.fr (Y.C.); a-merle@hotmail.fr (A.M.); 2 Laboratoire de l’Eau et de l’Environnement FSEA BP 1027 Université de N’Djamena, Chad; tnewmahamat@yahoo.fr

**Keywords:** Chari River, heavy metals, pollution, water surface, data analysis

## Abstract

This study was carried out to identify and assess the water quality of the Chari River. The Chari, 1200 km long, is Chad’s major water source. Municipal sewage, industrial wastewater discharge, and seasonal run-off from agriculture are regularly fed into the river. Several trace metals such as Cu, Zn, Fe, Ni, Cr, Mn, and Cd, were measured in different sampling stations located along the Chari River at N’Djamena in different campaigns from 2008 to 2010. Overall, manganese, zinc, chromium, and copper concentration levels were mainly in the range of the permissible limits prescribed by WHO guidelines (WHO 2011). Nickel, iron, and cadmium concentrations were still high. This preliminary study allowed us to identify the magnitude of toxic pollutants, which are responsible for Chari River water contamination in the study area. This study revealed that urgent measures must be taken to protect the local people from health problems resulting from high concentrations of heavy metals.

## 1. Introduction

Problems of water supply in urban areas in developing countries [1] require attention in order to guarantee the water quality of rivers and lakes. One such ecosystem in Africa is the Lake Chad Basin, with the Logone River and the Chari River. Research studies describing the water quality of the Logone River have been published [2,3]. Along this line of this study, we focused our attention on heavy metal pollution in the Chari River at N’Djamena.

Polluted water has direct effects on human health. Sewerage and industrial effluents have indirect effects through consumption of foodstuffs being irrigated with contaminated water. According to World Health Organization [4], more than 80% of human diseases have their origins in water [5]. Water pollutants mainly consist of heavy metals, microorganisms, fertilizers, and thousands of toxic organic compounds [4]. Heavy metals consist of Cd, Cr, Cu, Ni, Fe, Mn, and Zn as well as metals of groups III and IV, which have toxic effects on human physiology. Surface and ground waters also contain bacteria, protozoa, and other synthetic microorganisms. Contaminated water is also responsible for disease epidemics in human beings [6].

The presence of toxic metals at high and abnormal concentrations compared to natural loads is becoming an alarming problem in rivers. The intensification of anthropogenic pressure (industry and agriculture) may lead to disastrous effects due to the increased concentration of certain chemical substances above natural levels [7]. In Chad, several branches of industry such as agriculture, human health, breeding, industry, and cosmetics use significant quantities of various chemical products [8]. Massive and uncontrolled chemical-based production is the principal source of water pollution, leading to considerable damage to the environment and water. Agriculture is the main source of water pollution by nitrates and phosphates. It is therefore necessary to study the effect of these irrigation activities on the water quality of the Chari River. In addition to pollution, there have been issues of desertification and dryness (1972–1973 and 1984–1985) which prevailed in tropical Africa to the south of the Sahara [9,10,11].

No information is available on this deterioration of water quality. There is an urgent need to assess the water quality of the Chari River for proper conservation and efficient utilization of freshwater for sustainable development. Here, this preliminary study aims at assessing the present water quality of the Chari River with respect to agricultural and industrial activities in N’djamena. In addition, we focused our attention in the measurement and the quantification of heavy metals such as copper, manganese, zinc, iron, chromium, nickel, and cadmium. It was very important to quantify some of the heavy metals in water of the Chari River. The heavy metals assessed are copper, manganese, zinc, iron, chromium, nickel, and cadmium.

## 2. Materials and Methods

### 2.1. Overview of the Study Area

**Figure 1:**
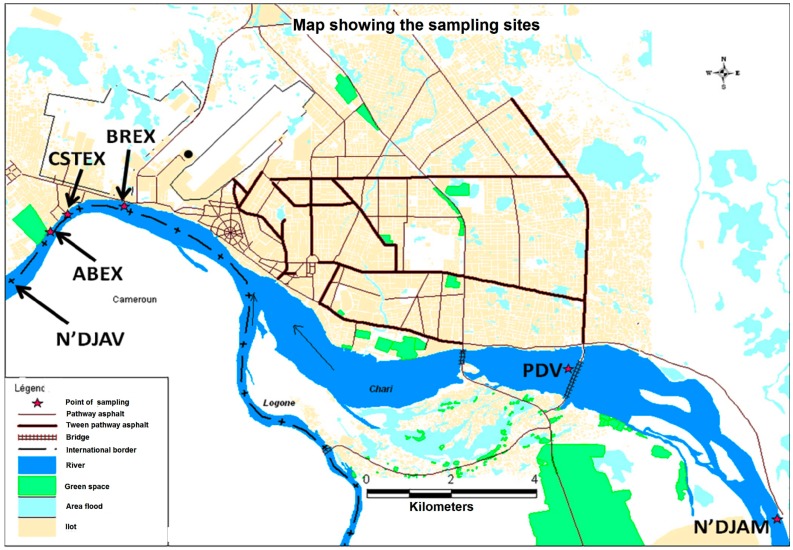
Map of N’Djamena showing the sampling sites: PDV: The bridge in Ndjamena; BREX: Discharge from the Chad breweries (BR). The Chad breweries are located 20 km from the PDV; CSTEX: Discharge from the national Chad Sugar Company (CST). It is located 2 km from BREX; ABEX: Discharge from the slaughterhouse/modern meatpacking plant. It is located 2 km from CSTEX; ABAV: Site located 75 m downstream of the slaughterhouse discharge.

The study was performed in the navigable part of the Chari River in N’Djamena (Chad) (Figure 1). The town of N’Djamena (1,100,000 inhabitants) is located on the right Chari riverside. N’Djamena is geographically located at latitude 12°8′ north of the equator and longitude 15°2′ east of the prime meridian. It is an administrative centre (university, industrial and commercial). The annual average temperature is 28 °C. The rain season (high water period) is spread out between May and September. It receives an average rainfall annually of 500 to 700 mm. The size of the Chari River is determined by rain and temperatures in the Sahel [11,12].

The Chad’s climate is controlled by two strong air masses. There is a dry continental air mass and a maritime air mass which sometimes collide, creating unexpected weather patterns [11]. In general, the depth of the air mass varies greatly throughout the year, causing unpredictable weather conditions. Precipitation takes place when a great depth of humid air mass accumulates in the Chad basin. The pluviometry data obtained by the national weather service has shown a heavy rainy period (the monsoon rains) in June, July, and August and a longer dry period which characterizes the Sahelian climate from October to May [12,13]. The Chari water level is sensitive to climate change [14].

Much of Chad’s population is concentrated around the Chari River. It provides 90% of the water flowing into Lake Chad [15]. The watershed of the river covers 548.747 km^2^. There is a strong relationship between human activity and pollution of the environment. The recognition of this connection and the need to protect human health, recreation, and fishery production led to the early development of water quality regulations and monitoring methods.

### 2.2. Sampling

A research program thus allowed us to set up, at the level of the discharge system of the industry effluents, a sampling device to study some chemical parameters. Metallic elements such as zinc, copper, manganese, iron, chromium, nickel, and cadmium were measured, at various periods of the year in the neighbourhoods of N’Djamena. The choice of sampling sites is a capital operation that determines the analytical results. After preliminary study of the physicochemical parameters, two different criteria were used to select the location of the sampling stations: a criterion based on geographical considerations and another on the activities developed in the riversides. Thus, water samples were collected using a probe in mid-stream at depths of 0.5 m. All samples were stored into clean plastic bottles at 4 °C and transported to laboratory refrigerator for further analysis. Samples were collected at least three times per sampling point for the characterization of water river variations during the sampling period.

Different sampling sites (Figure 1) were chosen along the Chari River, which included the bridge at N’djamena and three sites at each industry location. A description of different sampling sites is presented in Table 1. Sampling expeditions were performed in October, November, and December 2008, and January, July, August, and September 2009. The sampling covered both dry and wet seasons.

### 2.3. Analytical Procedures

The concentrations of heavy metals, except cadmium, were determined by spectrophotometer HACH DR 2400 [16]. The cadmium concentrations were measured using the spectrophotometer HACH DR 2800. The HACH analyser has several advantages, such as small volume, convenient transport, simple and rapid operation, low reagent consumption, and rapid measurement. It can be transported to the site. However, the use of the adopted standard reagents was limited by cost.

Forms of copper in water can be classified as insoluble, dissolved (free and complexed), and total recoverable. Insoluble copper includes precipitates such as copper sulphides and hydroxides. All copper in solution is known as dissolved copper, including Cu^1+^ (cuprous) and Cu^2+^ (cupric) ions and copper chelates such as Cu EDTA. Hach procedures used the porphyrin method (Hach, 2400). This method for determining copper is a very sensitive test, capable of detecting free copper (Cu^1+^ and Cu^2+^) and total recoverable copper (with digestion) in the range of 0–150 μg/L. Due to the sensitivity of the method, a blank value with high water quality was difficult to obtain. The porphyrin method used a split sample. One half of the split sample was treated with a masking agent to complex the free copper forms; then, the porphyrin reagent, a buffer, and a reducing agent was added. We obtained a “zero blank” without the need for special copper-free water. Porphyrin reagent was added to the second half of the split sample, where it reacted with the free copper. Interference caused by the reaction of porphyrin with other metals was minimized by using the split sample. Porphyrin reacted slowly with Cu^2+^. A special formulation of the porphyrin and the addition of a buffer allowed the reaction with free copper to be completed within seconds. A reducing agent was also added to destroy unreacted porphyrin (which would otherwise interfere). An intense absorbance at 425 nm makes this method very sensitive when using a colorimeter or spectrophotometer. Traces of copper were found in all the water samples analysed with the highest concentrations of 0.19 mg/L at the ABAV site and 0.25 mg/L at the BREX site, respectively. The primary source of copper contamination in drinking water is the corrosion of copper pipes or fittings.

The Hach method used for the determination of zinc concentrations was the ZincoVer^®^ method. This method allowed measurements of zinc concentration ranges between 0.01 and 2.00 mg/L. In the analysis of zinc, cyanide was added to a buffered water sample of pH 9 to form a complex with all heavy metals present in the sample. The addition of cyclohexanone then freed zinc from the cyanide complex and enabled it to react with the indicator (zincon, a dry powder form of 2-carboxy-2′hydroxy-5′sulfoformazyl benzene). A blue-coloured complex formed in direct proportion to the amount of zinc in the sample. The measurement of the colour intensity at 620 nm determined the zinc concentration.

The method used for colorimetric iron analysis in Hach procedures was the 1,10-phenanthroline method. It is the best-known test for iron. The Fe^2+^ procedure used Ferrous Iron Reagent Powder containing 1,10-phenanthroline as an indicator. Total iron determination or analysis was performed using FerroVer Iron Reagent. The FerroVer Iron Reagent contained 1,10-phenanthroline, combined with a reducing agent, to convert all but the most resistant forms of iron present in the sample to Fe^2+^. The 1,10-phenanthroline contained in the Ferrous Iron Reagent Powder reacted with Fe^2+^ to form a characteristic orange-coloured complex. The intensity of colour development was directly proportional to the amount of Fe^2+^ in the sample. Total iron also can be determined with the FerroVer Iron Reagent. This method allowed iron analysis in the range of 0.02 to 3 mg/L at 510 nm.

For nickel analysis, the 1-(2-pyridylazo)-2-naphthol (PAN) method was used. The PAN procedure is a very sensitive method for detecting nickel in concentrations of less than 1 mg/L. The method is relatively free from interference. It can be made on the same sample portion without the need for solvent extraction or sample preconcentration steps. PAN was suspended in water by use of surfactants to allow it to form complexes with the metals in the sample. A complexing agent can be used to decompose all PAN chelates except those of nickel. A pH adjustment using the Phthalate-Phosphate Reagent enhances the rate of development of the coloured nickel PAN complexes. The absorbance of the cobalt PAN complex at 560 nm is the same as at 620 nm; however, absorbance caused by the nickel PAN complex is zero at 620 nm. This difference in absorbance wavelengths allowed nickel to be determined without interference by measuring the absorbance at 560 nm and subtracting the absorbance at 620 nm.

In the analysis for total chromium, the sample was heated to the boiling point under strong alkaline conditions in the presence of hypobromite. The trivalent chromium was converted to hexavalent chromium. The proper chemical conditions for this oxidation were provided by Chromium 1 Reagent Powder. After oxidation completion, excess hypobromite was destroyed by the addition of Chromium 2 Reagent Powder. Then, ChromaVer 3 Chromium Reagent, which contains an acidic buffer combined with 1,5-diphenylcarbohydrazide, was added. A purple color developed with intensity directly proportional to the total chromium concentration. The absorbance was measured at 540 nm.

The 1-(2-pyridylazo)-2-naphthol (PAN) method is a sensitive, rapid procedure for low levels of manganese. The PAN method uses Alkaline Cyanide Reagent to mask interferences. PAN Indicator was added and formed an orange-red colored complex with the manganese ions present. The absorbance was measured at 560 nm.

The cadmium concentrations were determined by the dithizone method, allowing the measurement of a range of concentrations from 0.0007 to 0.08 mg/L. Cadmium ions in sample solution react with dithizone to form a pink to red cadmium–dithizonate complex, which is extracted with chloroform. Test results were measured at 515 nm.

### 2.4. Data Analysis

Principal component analysis (PCA) [17,18] was performed on all the results. The aim of PCA is to reduce the dimensionality of a dataset by building a new space where each axis (principal component or PC) is a linear combination of all the initial variables. PCA allows us to structure and summarize variables and individuals to better understand the information provided by the variables. PC are calculated to be included in the variance of in the dataset [17]. Ideally, a limited number of PCs allows a reliable representation of the initial data. All PCA were performed on SAS software: Jump 10.

## 3. Results

The concentration average of the seven heavy metals studied here are presented in Table 1. These concentrations represent means of all data collected in this study at every sampling site.

### 3.1. Copper

**Figure 2:**
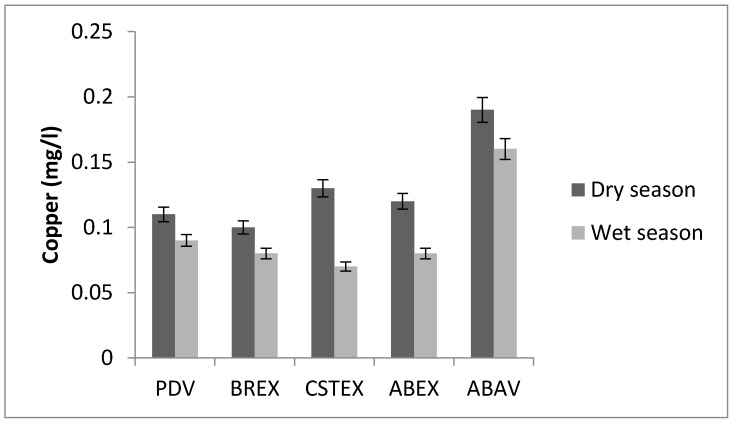
Copper concentration.

Copper concentrations in potable water are usually very low. The copper concentrations in water samples were in the range of 0.07–0.17 mg/L (Figure 2). Copper may occur in natural waters, wastewaters, and industrial waste streams as soluble copper salts or as copper compounds precipitated on suspended solids. Copper is not considered as a health hazard to humans. More than 1 mg/L can impart a bitter taste to water and large oral doses can cause vomiting and may eventually cause liver damage [18].

### 3.2. Zinc

**Figure 3:**
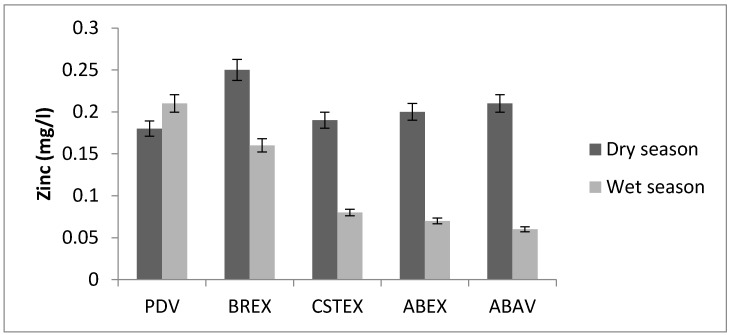
Zinc concentration.

Traces of zinc were found in all the water samples analysed with the highest concentrations of 0.19 mg/L at the ABAV site and 0.25 mg/L at the BREX site, respectively (Figure 3).

The limit described by the WHO guideline for Zn is 5 mg/L. Industrial effluents may contribute to large amounts of zinc. Zinc is essential to human metabolism and has been found to be necessary for proper growth. Consumption of water contaminated with high concentrations of zinc may lead to stomach irritation, vomiting, depression, malaise and cough, but these effects are temporary [3]. Concentrations above 5 mg/L show no harmful physiological effects but can cause a bitter taste and/or opalescence in alkaline drinking water.

### 3.3. Iron

**Figure 4:**
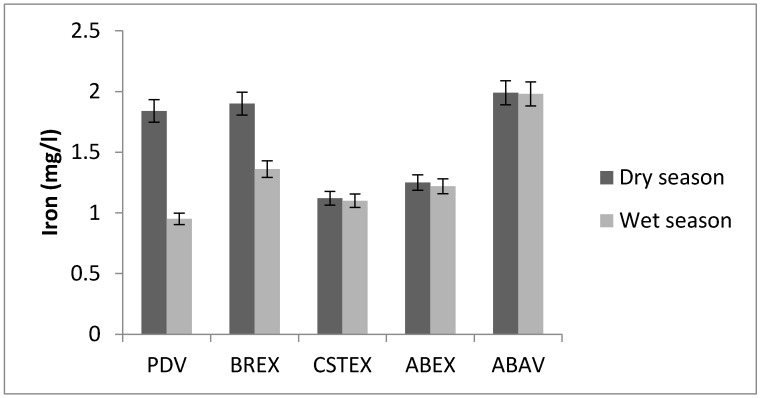
Iron concentration.

Iron is the most abundant element in the waters of the Chari. Iron concentration in water samples was in the range between 0.90 and 1.90 mg/L (Figure 4). The highest concentrations were recorded during the sampling in January 2010: PDV, BREX, ABAV. These values are higher than in the WHO recommendations [4].

### 3.4. Nickel

**Figure 5:**
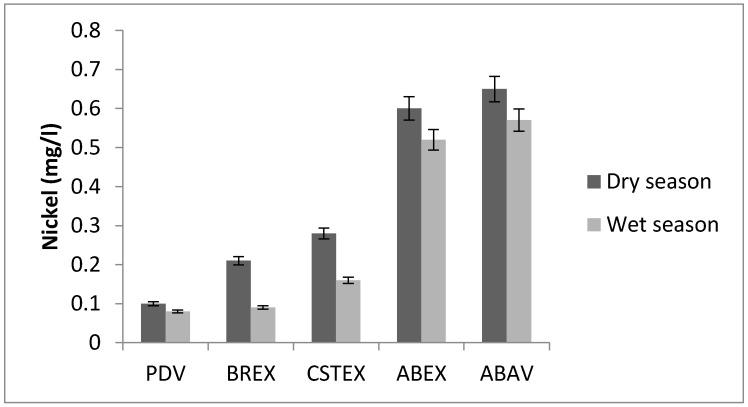
Nickel concentration.

The nickel concentrations in water samples were in the range between 0.09 mg/L (PDV) and 0.65 mg/L (ABAV) (Figure 5). The concentrations of nickel (Figure 5) increased at the level of slaughterhouse discharge (ABEX and ABV sites). The ABEX and ABAV levels are six times higher than the mean values of the values recorded at the PDV points. These sites are an indicator of excessive nickel intake.

### 3.5. Chromium

**Figure 6:**
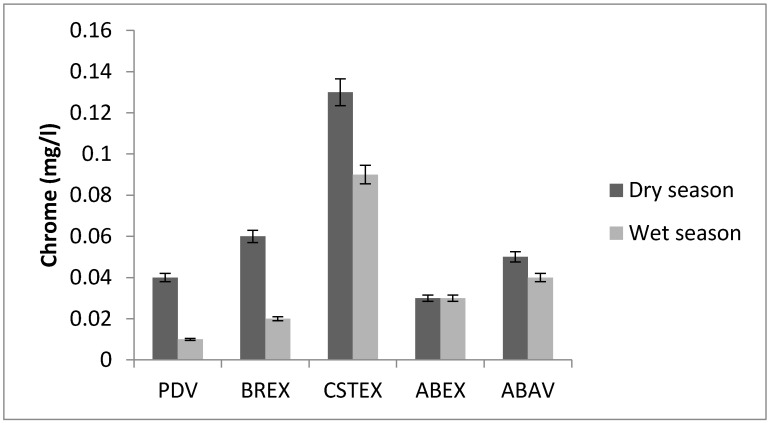
Chromium concentration.

The chromium concentrations in water samples were in the range between 0.02 mg/L and 0.13 mg/L (Figure 6). The maximum level was recorded in the effluent of the landfill at the CSTEX station. Chromate is used to inhibit metal corrosion. Chromium is an unwanted contaminant in public drinking water supplies due to its suspected carcinogenic effects [19]. Chromium present in potable waters at a level above 0.003 mg/L indicates the possible presence of industrial wastes. Chromium concentrations greater than 0.05 mg/L are sufficient grounds to reject the water supply. With respect to WHO guidelines (0.05 mg/L for Cr), the mean concentrations of chromium are slightly lower (Figure 6), whereas manganese concentrations are slightly higher on average.

### 3.6. Manganese

**Figure 7:**
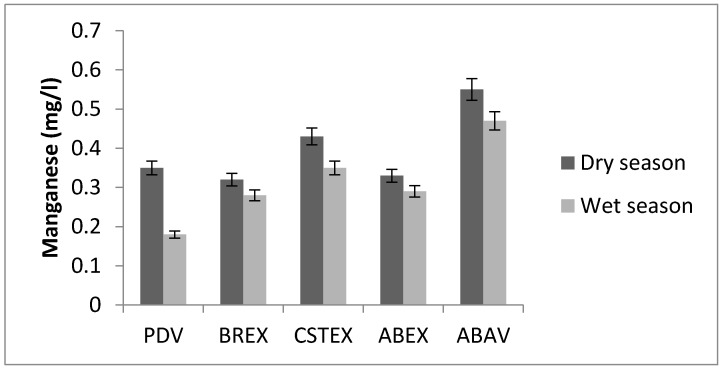
Manganese concentration.

The amount of manganese in water of the Chari River varied between 0.18 mg/L and 0.55 mg/L, with the maximum level found at the ABAV station (Figure 7). These concentrations were on average slightly below the permissible limits of WHO guidelines (0.4 mg/L for Mn). Manganese is a very common compound that can be found all over Earth. Manganese that derives from human sources can also enter surface water, groundwater, and sewage water through the application of manganese pesticides. Manganese is one of three toxic essential trace elements, which means that it is not only necessary for humans to survive, but it is also toxic when too high concentrations are present in a human body. Manganese levels in natural waters rarely exceed 1 mg/L, but levels of 0.1 mg/L are sufficient to cause the taste and staining problems [3].

### 3.7. Cadmium

**Figure 8:**
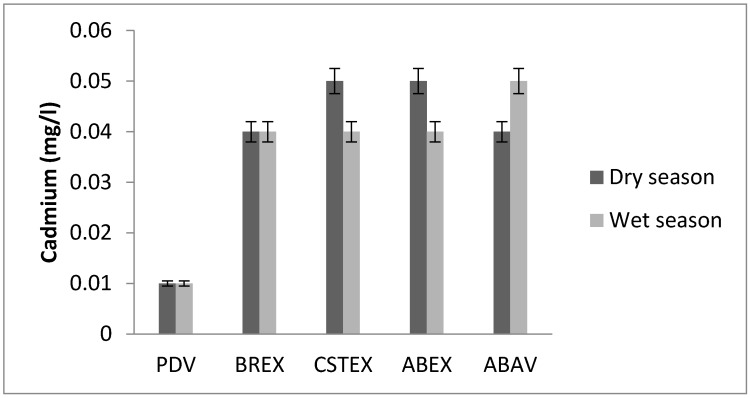
Cadmium concentration.

Cadmium concentration in water samples was in the range between 0.01 and 0.05 mg/L (Figure 8) [20]. These levels vary from one season to the next. These values are much higher than the guideline value set by the WHO [4].

### 3.8. Principal Component Analysis (PCA)

In this part of the study, the data are expressed in the flux of materials that integrates flows.

For the correlation matrix (Table 2), there is a correlation between two vectors x and y if:

- Positive correlation: x and y vary in the same direction
- Negative correlation: x and y vary in the opposite direction of the other
- No correlation: x and y vary independently

In our case, metals are correlated with one to another and their quantities vary generally in the same way. With the large number of measurements, the correlation is statistically significant for a correlation coefficient greater than 0.75 in absolute value (according the Pearson Table).

In order to determine statistically the effect of this pollution on the sites, we applied the PCA on all the data obtained during the sampling campaigns. In this part of the study, the data are expressed in the flux of materials that integrates flows.

The study carried out shows that the three components can explain nearly 64% of the observed variations. With four components, this figure reached 74%.

**Figure 9:**
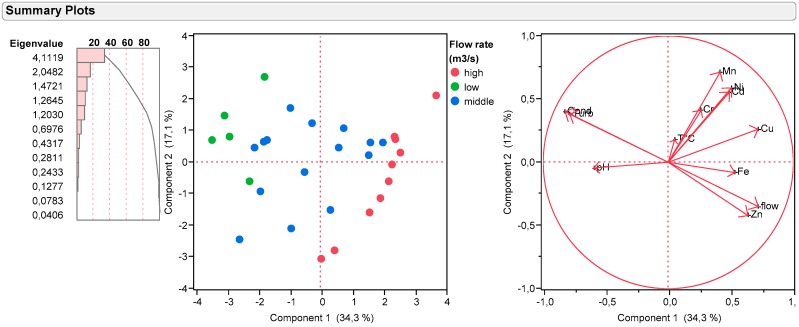
Summary plot scores and loadings plot.

As a first step, individuals are labelled according to river flows (Figure 9). In this case, we observe a partition into three distinct groups that are differentiated by flow. High flow rates are characterized by lower pH, lower conductivity, and higher metal fluxes. An interesting way to differentiate the type of metal pollution of the river is to take flows into account. The information obtained needs to be supplemented with other samples. In the future, an automatic flow measurement system could be coupled by taking samples analysed. These studies will be carried out with a complete set of data: no points would be deleted to perform the data analysis. The results suggest that the source of pollution is identical when the variables are highly correlated (Cu and Ni).

## 4. Discussion

The mean values of the elements at different zones in the Chari River showed iron, nickel and cadmium as the most abundant elements. These elements are the most important water contaminants in the Chari River [21,22,23]. The nickel concentrations were the highest at the ABAV site (0.65 mg/L) (Figure 5). The nickel concentration maximum at the ABAV site and the nickel in drinking water was less than 0.3 mg/L and 0.07 mg/L, respectively [3]. Finally, the abnormally high cadmium concentrations ranged from 0.01 to 0.05 mg/L [6], ten times above the recommended levels of the WHO guidelines (i.e., 0.003 mg/L). Generally, these concentrations increased in the Chari water from the N’Djamena upstream to downstream. The upstream/downstream variation may be attributed to agricultural and industrial activities dealing with engineering goods, chemicals. Excess consumption of non-essential metals such as Cd results in neurological, bone, and cardiovascular diseases, renal dysfunction, and various cancers, even at relatively low levels [24]. The study carried out on the river Logone [25] shows Cd levels 10 times lower in water (5 × 10^−3^ mg/L against 5 × 10^−2^ mg/L); a rate too high according to the WHO (maximum 0.003 mg/L).

Iron is one of the most persistent elements in water supplies. Natural waters contain variable but minor amounts of iron, despite its universal distribution and abundance. Iron can enter a water system from leaching of natural deposits, iron-bearing industrial wastes, or from effluents of pickling operations. Excess of iron in water gives distasteful taste. Iron is not hazardous to health, but is considered as a secondary or aesthetic contaminant [3]. Water used in industrial processes must contain less than 0.2 mg/L of total iron [21].

Nickel is seldom found in natural waters, but is often present in industrial wastewater as a direct product of metal plating baths, and as a corrosion product of stainless steel, nickel alloys. Nickel is considered relatively nontoxic to humans. The toxicity of nickel to aquatic life varies widely and is influenced by species, pH, synergetic effects, and other factors [20,21,22,23,24,25,26,27]. The nickel concentration increases from upstream to downstream with a very high value for ABAV, well above the recommended values for drinking water (0.3 to 0.07 mg/L) [3].

From the analysis of the data obtained for all the sampling campaigns, the results (Table 3) show proficiency classes that vary from good to bad class for the Chari River. The absence of treatment of wastewater from the discharge results in high levels of these metals. The origin of heavy metal contamination can be linked to different inputs: wastewater discharge from domestic sources; industrial waste water; wastewater discharge from hospital sources; discharges related to the maintenance of road vehicles. These observations reveal an important contribution of the anthropogenic activities. At the PDV point, the situation is slightly degraded, as this station is located upstream of the main discharge points (industrial outlets and other spill points). Chari water quality monitoring shows that all water discharge points in the river exceed the WHO quality standards with respect to more than half of the parameters analyzed. These observations reveal that Chari water is unsuitable for drinking. From the data analysis of this study, irrigation use is possible. However, the constraints imposed are less drastic, meaning that the quality of the water obtained from the Chari River can be described as good with respect to the SEQ-Eau [23].

Less vulnerable animals (sheep and cattle) can drink under supervision and away from wastewater spills. The metallic quality of the Chari and more particularly the cadmium and lead contents do not allow for good drinking water for the animals. Authors should discuss the data and how the results can be interpreted in the perspective of future studies. The findings and their implications should be discussed in the broadest context possible. Future research directions may also be highlighted.

The environment of the Chari River at the sites analyzed has not changed at all since 2010, as no treatment has been put in place to decontaminate the discharge. New campaigns to analyze heavy metals, fauna, and flora would further confirm this pollution.

## 5. Conclusions

Metals are natural constituents of the Earth’s crust that can spread naturally in the aquatic environment. Due to anthropogenic activities pressures (industry and agriculture), heavy metal contamination of aquatic environments can increase significantly. The different results obtained highlight that the average of metallic elements increases from upstream to downstream, due to the pollution load from industrial, agricultural, and domestic discharge. Heavy metals concentrations at the industrial outlets are high, but the level of dilution is also important. All the metals metered are located at the industrial effluents and the Chari River. Analysis of fauna and flora placed in the vicinity of the Chari River and effluents would further confirm this pollution.

## Figures and Tables

**Table 1:** Mean metal values from the sampling points.

	Mean Values									
	Dry Weather 2008					Rainy Weather 2009				
Parameters	PDV	BREX	CSTEX	ABEX	ABAV	PDV	BREX	CSTEX	ABEX	ABAV
**Zn (mg/L)**	0.18	0.25	0.19	0.20	0.21	0.21	0.16	0.08	0.07	0.06
**Cu (mg/L)**	0.11	0.10	0.13	0.12	0.19	0.09	0.08	0.07	0.08	0.16
**Cr (mg/L)**	0.04	0.06	0.13	0.03	0.05	0.01	0.02	0.09	0.03	0.04
**Mn (mg/L)**	0.35	0.32	0.43	0.33	0.55	0.18	0.28	0.35	0.29	0.47
**Ni (mg/L)**	0.10	0.21	0.28	0.60	0.65	0.08	0.09	0.16	0.52	0.57
**Fe (mg/L)**	1.84	1.90	1.12	1. 25	1.99	0.95	1.36	1.10	1.22	1.98
**Cd (mg/L)**	0.01	0.04	0.05	0.05	0.04	0.01	0.04	0.04	0.04	0.05

**Table 2:** Correlation matrix.

Row	Flow	T, °C	pH	Cond	Turb	Cd	Zn	Cu	Cr	Mn	Ni	Fe
flow	1.00	0.35	−0.50	−0.69	−0.59	0.24	0.44	0.30	0.09	−0.04	0.08	0.43
T °C	0.35	1.00	−0.16	0.12	0.21	0.13	−0.18	0.12	−0.06	−0.04	0.09	0.07
pH	−0.50	−0.16	1.00	0.44	0.24	−0.25	−0.15	−0.51	−0.19	−0.11	−0.20	−0.29
Cond	−0.69	0.12	0.44	1.00	0.89	−0.09	−0.59	−0.55	−0.12	−0.13	−0.17	−0.24
Turb	−0.59	0.21	0.24	0.89	1.00	−0.17	−0.64	−0.52	−0.16	−0.12	−0.16	−0.22
Cd	0.24	0.13	−0.25	−0.09	−0.17	1.00	0.20	0.17	0.54	0.43	0.46	0.36
Zn	0.44	−0.18	−0.15	−0.59	−0.64	0.20	1.00	0.23	0.00	0.08	0.05	0.52
Cu	0.30	0.12	−0.51	−0.55	−0.52	0.17	0.23	1.00	0.12	0.55	0.56	0.15
Cr	0.09	−0.06	−0.19	−0.12	−0.16	0.54	0.00	0.12	1.00	0.35	−0.03	−0.12
Mn	−0.04	−0.04	−0.11	−0.13	−0.12	0.43	0.08	0.55	0.35	1.00	0.58	0.03
Ni	0.08	0.09	−0.20	−0.17	−0.16	0.46	0.05	0.56	−0.03	0.58	1.00	0.40
Fe	0.43	0.07	−0.29	−0.24	−0.22	0.36	0.52	0.15	−0.12	0.03	0.40	1.00

**Table 3:** Metal quality classes (mean analytical results expressed in mg/L) as a function of the flow levels of the Chari River for each site.

Location	Flow (m^3^/s)	Cd	Pb	Zn	Cu	Cr	Mn	Ni	Fe
**PDV**	High	0.02	0.02	0.16	0.13	0.02	0.10	0.01	1.84
	Middle	0.01	0.02	0.20	0.10	0.01	0.20	0.13	0.80
	Low	0.01	0.01	0.20	0.08	0.03	0.40	0.12	1.49
**BRAM**	High	0.04		0.11	0.11	0.03	0.11	0.01	1.47
	Middle	0.04		0.08	0.06	0.02	0.25	0.20	1.16
	Low	0.03	0.03	0.03	0.06	0.07	0.03	0.50	0.15
**BREX**	High	0.04	0.07	0.27	0.13	0.06	0.30	0.20	2.02
	Middle	0.04	0.05	0.21	0.09	0.03	0.30	0.15	1.56
	Low	0.04	0.03	0.16	0.07	0.05	0.30	0.11	1.42
**BRAV**	High	0.04		0.16	0.23	0.01	0.25	0.02	1.48
	Middle	0.03	0.03	0.01	0.07	0.07	0.01	0.25	0.08
	Low	0.03	0.03	0.08	0.14	0.04	0.37	0.17	1.66
**CSTAM**	High	0.03		0.09	0.13	0.03	0.23	0.04	1.46
	Middle	0.02		0.04	0.12	0.04	0.11	0.05	0.35
	Low	0.03		0.28	0.08	0.07	0.22	0.52	0.33
**ABAM**	High	0.04		0.20	0.10	0.03	0.40	0.11	1.87
	Middle	0.06	0.06	0.08	0.11	0.03	0.35	0.94	1.21
	Low	0.05	0.03	0.10	0.11	0.03	0.43	0.71	1.69
**ABEX**	High	0.06	0.06	0.22	0.13	0.02	0.34	0.59	2.58
	Middle	0.05	0.03	0.16	0.10	0.04	0.31	0.58	1.87
	Low	0.04	0.02	0.09	0.10	0.03	0.29	0.53	1.67
**ABAV**	High	0.05	0.07	0.21	0.20	0.06	0.60	0.68	2.23
	Middle	0.04	0.04	0.15	0.17	0.04	0.55	0.58	1.62
	Low	0.04	0.03	0.11	0.17	0.05	0.43	0.59	1.40

□ (white color): Good quality water; □ (yellow color): Significant degradation from natural state. Medium quality water; □ (orange color): Significant degradation from natural state. Poor quality water; □ (red color): Degradation very important in relation to the natural state. Poor quality water.
